# Photoswitchable Fluorescent Diarylethene Derivatives with Thiophene 1,1-Dioxide Groups: Effect of Alkyl Substituents at the Reactive Carbons

**DOI:** 10.3390/ma10091021

**Published:** 2017-09-02

**Authors:** Masakazu Morimoto, Takaki Sumi, Masahiro Irie

**Affiliations:** Department of Chemistry and Research Center for Smart Molecules, Rikkyo University, 3-34-1 Nishi-Ikebukuro, Toshima-ku, Tokyo 171-8501, Japan; 13lb003x@rikkyo.ac.jp

**Keywords:** photoswitch, diarylethene, photochromism, fluorescence, super-resolution fluorescence microscopy

## Abstract

Photoswitching and fluorescent properties of sulfone derivatives of 1,2-bis(2-alkyl-4-methyl-5-phenyl-3-thienyl)perfluorocyclopentene, **1**–**5**, having methyl, ethyl, *n*-propyl, *i*-propyl, and *i*-butyl substituents at the reactive carbons (2- and 2′-positions) of the thiophene 1,1-dioxide rings were studied. Diarylethenes **1**–**5** underwent isomerization reactions between open-ring and closed-ring forms upon alternate irradiation with ultraviolet (UV) and visible light and showed fluorescence in the closed-ring forms. The alkyl substitution at the reactive carbons affects the fluorescent property of the closed-ring isomers. The closed-ring isomers **2b**–**5b** with ethyl, *n*-propyl, *i*-propyl, and *i*-butyl substituents show higher fluorescence quantum yields than **1b** with methyl substituents. In polar solvents, the fluorescence quantum yield of **1b** markedly decreases, while **2b**–**5b** maintain the relatively high fluorescence quantum yields. Although the cycloreversion quantum yields of the derivatives with methyl, ethyl, *n*-propyl, and *i*-propyl substituents are quite low and in the order of 10^−5^, introduction of *i*-butyl substituents was found to increase the yield up to the order of 10^−3^. These results indicate that appropriate alkyl substitution at the reactive carbons is indispensable for properly controlling the photoswitching and fluorescent properties of the photoswitchable fluorescent diarylethenes, which are potentially applicable to super-resolution fluorescence microscopies.

## 1. Introduction

Fluorescent molecules with photoswitching ability have attracted much attention because of their potential applications to optical information storage as well as super-resolution fluorescence microscopies [1,2,3,4,5]. One methodology to construct the photoswitchable fluorescent molecules is to integrate both photochromic and fluorescent chromophores in a molecule [6,7,8,9,10,11,12,13,14,15,16]. These molecules are initially fluorescent while the fluorescence is quenched by an energy or an electron transfer mechanism when the photochromic unit undergoes isomerization upon photoirradiation. The turn-off mode fluorescence photoswitching can be applied to optical information storage but is hardly applicable to super-resolution fluorescence microscopies, such as PALM (photoactivatable localization microscopy) and STORM (stochastic optical reconstruction microscopy), because these imaging techniques require a dark background to detect single fluorescent molecules [17,18,19,20,21]. For these applications, it is strongly desired to develop turn-on mode photoswitchable fluorescent molecules which can be efficiently and instantaneously activated upon photoirradiation.

Recently, a new type of turn-on mode photoswitchable fluorescent molecules, sulfone derivatives of 1,2-bis(benzothiophenyl)perfluorocyclopentene, has been developed [22,23,24,25,26,27,28,29,30,31,32,33,34]. The first example is the sulfone derivative of 1,2-bis(2-methyl-1-benzothiophen-3-yl)perfluorocyclopentene, which was reported by Ahn and co-workers [22]. Upon irradiation with UV light, the open-ring isomer undergoes a cyclization reaction to produce the fluorescent closed-ring isomer. However, the fluorescence quantum yield is relatively low (Φ_f_ = 0.011). In order to improve the fluorescent property, various chemical modifications have been carried out. It was found that the fluorescence quantum yield depends on substituents at 2- and 2′- and 6- and 6′-positions of the benzothiophene 1,1-dioxide groups. Introduction of phenyl groups at the 6- and 6′-positions dramatically increased the fluorescence quantum yield of the closed-ring isomer up to 0.64 [27,34]. The yield was further increased up to 0.9 by replacing the methyl substituents at the 2- and 2′-positions with short alkyl chains, such as ethyl, *n*-propyl, and *n*-butyl groups [26,27,34]. Very recently, it has been demonstrated that such turn-on mode fluorescent diarylethene derivatives can be utilized as molecular probes for super-resolution fluorescence microscopies, such as PALM/STORM [35,36,37] and RESOLFT (reversible saturable (switchable) optical linear fluorescence transitions) microscopy [38]. 

**Scheme 1:**
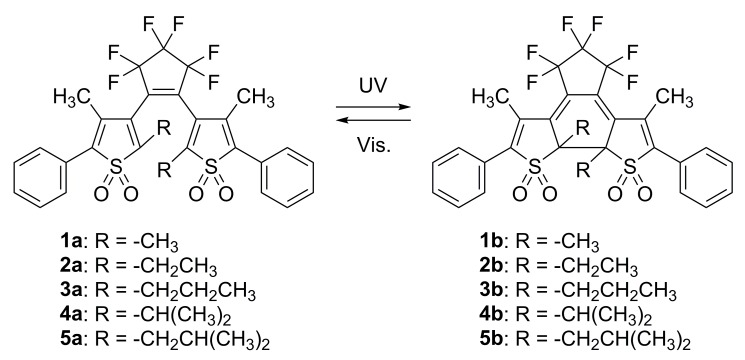
Photoisomerization of photoswitchable fluorescent diarylethenes **1**–**5**.

A sulfone derivative of 1,2-bis(2,4-dimethyl-5-phenyl-3-thienyl)perfluorocyclopentene (**1**) has also been reported to exhibit fluorescence photoswitching (Scheme 1) [39]. Although the open-ring isomer **1a** undergoes a cyclization reaction upon UV irradiation to form the fluorescent closed-ring isomer **1b**, the fluorescence quantum yield of **1b** is relatively low (Φ_f_ = 0.03 in 2-methyltetrahydrofuran (2MeTHF)). In this paper, we synthesized four sulfone derivatives of 1,2-bis(2-alkyl-4-methyl-5-phenyl-3-thienyl)perfluorocyclonentene (**2**–**5**), as shown in Scheme 1, to improve the photoswitching and fluorescent properties. These derivatives possess different alkyl substituents, such as ethyl (**2**), *n*-propyl (**3**), *i*-propyl (**4**), and *i*-butyl (**5**) groups, at the reactive carbons (2- and 2′-positions) of the thiophene 1,1-dioxide rings. The effect of the alkyl substituents on the photoswitching and fluorescent properties has been studied.

## 2. Results and Discussion

**Figure 1:**
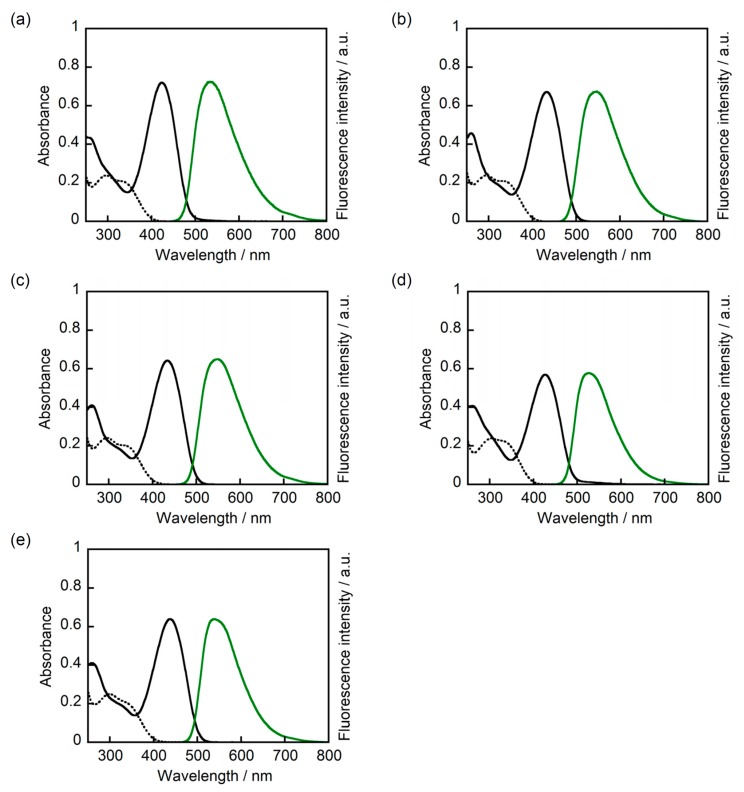
Absorption and fluorescence spectra of **1** (**a**); **2** (**b**); **3** (**c**); **4** (**d**); and **5** (**e**) in 1,4-dioxane (3.0 × 10^−5^ M). Black dashed lines: absorption spectra of open-ring isomers **1a**–**5a**, black solid lines: absorption spectra of closed-ring isomers **1b**–**5b**, green solid lines: fluorescence spectra (uncorrected) of closed-ring isomers **1b**–**5b**.

Figure 1 shows absorption spectra of the open-ring isomers **1a**–**5a** and absorption and fluorescence spectra of the corresponding closed-ring isomers **1b**–**5b** in 1,4-dioxane. **1a**–**5a** are colorless and have no optical absorption in the visible-wavelength region. Upon irradiation with UV (λ = 313 nm) light, **1a**–**5a** underwent cyclization reactions to produce the closed-ring isomers **1b**–**5b** and the 1,4-dioxane solutions turned yellow. The absorption spectra of **1b**–**5b** have maxima at around 430 nm and **1b**–**5b** emit green fluorescence at around 530 nm under irradiation with 430 nm light. The closed-ring isomers were thermally stable at room temperature in the dark. Upon irradiation with visible (λ > 440 nm) light, the absorbances and fluorescence intensities of **1b**–**5b** gradually decreased, indicating that the closed-ring isomers underwent cycloreversion reactions to form the open-ring isomers. Thus, **1**–**5** undergo photochromism and fluorescence photoswitching upon irradiation with UV and visible light. 

The effect of the alkyl substituents at the reactive carbons on the photoswitching and fluorescent properties was examined in more detail. Table 1 summarizes photophysical and photochemical parameters of diarylethenes **1**–**5** in 1,4-dioxane. The absorption spectra depend on the alkyl substituents at the reactive carbons to some extent. The absorption maxima of the open-ring isomers are located in the range of 296–301 nm. Appreciable difference among the five derivatives was not observed. On the other hand, the absorption maximum of the closed-ring isomer shows a significant bathochromic shift as much as 9 nm (from 424 nm to 433) when the substituent is changed from methyl (**1b**) to ethyl (**2b**). The absorption maximum of *n*-propyl substituted derivative **3b** (434 nm) is similar to that of ethyl substituted **2b**. *i*-Butyl substituted derivative **5b** shows a further bathochromic shift to 438 nm. The bathochromic shift upon *i*-propyl substitution is relatively small (**4b**: 427 nm). A similar spectral shift by alkyl substitution at reactive carbons is also observed for 1,2-bis(2-alkyl-5-phenyl-3-thienyl)perfluorocyclopentene [40]. The effect on fluorescence quantum yields (Φ_f_) is also noteworthy. The fluorescence quantum yield of the closed-ring isomer in 1,4-dioxane increases from 0.07 (**1b**) to 0.42 (**2b**) when the methyl substituents are replaced with the ethyl ones. The *n*-propyl, *i*-propyl, and *i*-butyl substituted derivatives also show high quantum yields of 0.42 (**2b**), 0.42 (**3b**), and 0.50 (**5b**), respectively, although these values are lower than those of sulfone derivatives of 1,2-bis(2-alkyl-6-phenyl-1-benzothiophen-3-yl)perfluorocyclopentene [26,27]. The fluorescence quantum yield of the closed-ring isomer is significantly improved by replacing the methyl substituents with the longer or branched alkyl ones. The geometrical or/and electronic structures of the closed-ring isomers are considered to be strongly influenced by the alkyl substitution at 2- and 2′-positions of the thiophene 1,1-dioxide rings, as observed in the sulfone derivatives of 1,2-bis(2-alkyl-6-phenyl-1-benzothiophen-3-yl)perfluorocyclopentene [27]. 

**Figure 2:**
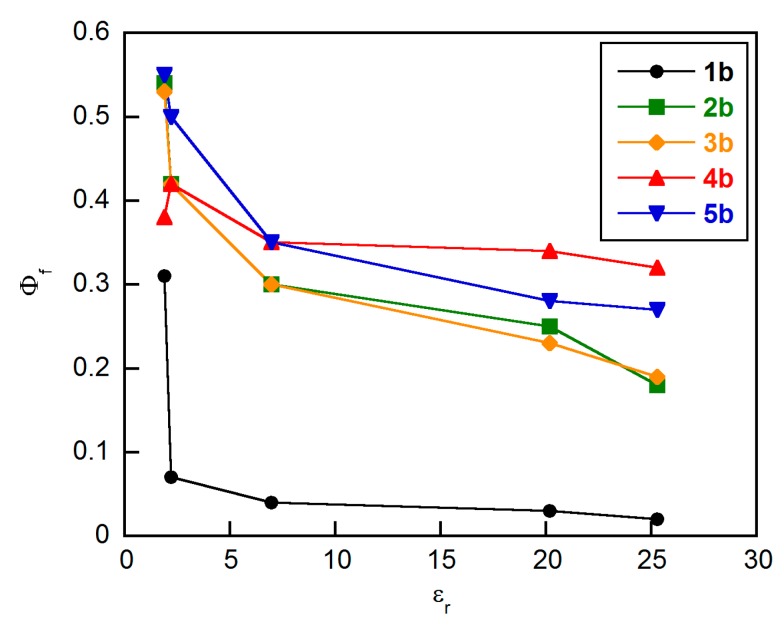
Relationship between fluorescence quantum yields (Φ_f_) of closed-ring isomers **1b**‒**5b** and the relative dielectric constants (ε_r_) of the solvents. The solvents used were *n*-hexane (ε_r_ = 1.89), 1,4-dioxane (ε_r_ = 2.22), 2MeTHF (ε_r_ = 6.97), 2-propanol (ε_r_ = 20.2), and ethanol (ε_r_ = 25.3).

The fluorescence quantum yields of **1b**–**5b** were also measured in various solvents. Figure 2 shows the relationship between the quantum yield and the relative dielectric constant (ε_r_) of the solvents (also see Table S1 in the supplementary material). The solvents used are *n*-hexane, 1,4-dioxane, 2MeTHF, 2-propanol, and ethanol. Methyl substituted derivative **1b** shows the relatively high quantum yield of 0.31 in nonpolar *n*-hexane, while the yield markedly decreases to 0.07 in 1,4-dioxane and further decreases to 0.04 in 2MeTHF, 0.03 in 2-propanol, and 0.02 in ethanol. In contrast, **2b**–**5b** show a different tendency. In *n*-hexane, **2b**–**5b** show higher fluorescence quantum yields (0.38–0.55) than **1b**. As the solvent polarity increases, the yields of **2b**–**5b** gradually decrease but keep relatively high values even in polar ethanol. In ethanol, the yields of the ethyl and *n*-propyl substituted derivatives (**2b** and **3b**) are 0.18 and 0.19, respectively. The derivatives with branched alkyl substituents show higher values (**4b**: 0.32, **5b**: 0.27). These results indicate that introduction of relatively large or branched alkyl substituents into the reactive carbons is effective to improve the fluorescent property in polar solvents. 

**Figure 3:**
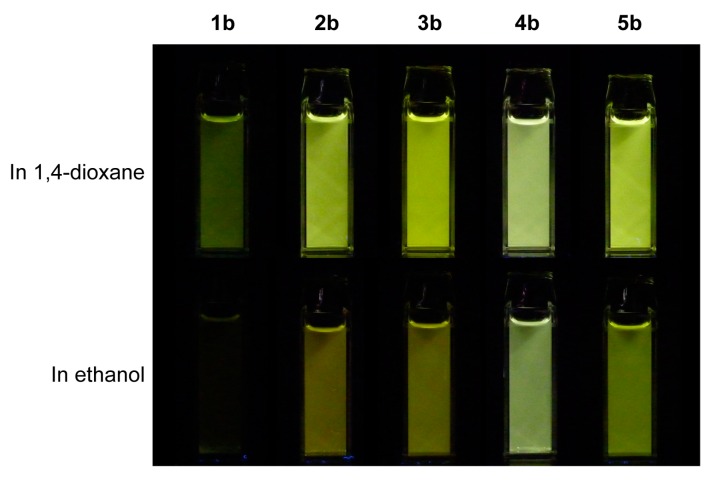
Photographs of fluorescent emissions of closed-ring isomers **1b**–**5b** in 1,4-dioxane and ethanol.

Figure 3 shows photographs of the fluorescent emissions of **1b**–**5b** in 1,4-dioxane and ethanol. The solutions of **2b**–**5b** show more bright emissions than that of methyl substituted **1b**. In ethanol, **1b** scarcely shows emission, while **2b**–**4b** still remain emissive. Such photoswitchable fluorescent molecules which maintain their fluorescent property even in polar environments are suitable for the application to bioimaging. 

Photocyclization and photocycloreversion quantum yields of **1**–**5** in 1,4-dioxane were also measured and the data are shown in Table 1. The cyclization quantum yields (Φ_oc_) of **1a**–**5a** are moderate and are almost in the range of 0.1–0.2. The alkyl substituents at the reactive carbons scarcely affect photocyclization reactivity. The cycloreversion quantum yields (Φ_co_) of **1b**–**4b** are in the order of 10^−5^, which are lower than those of the sulfone derivatives of 1,2-bis(2-alkyl-6-phenyl- 1-benzothiophen-3-yl)perfluorocyclopentene [26,27,34]. On the other hand, *i*-butyl substituted derivative **5b** shows a higher cycloreversion quantum yield of 2.1 × 10^−3^, which is 81 times larger than that of **4b**. Although the origin of the effect of *i*-butyl substitution is not clear at present, this result indicates that introduction of *i*-butyl substituents at the reactive carbons is useful for increasing the cycloreversion quantum yield. 

Photoactivatable or photoswitchable fluorescent molecules with extremely low switching-off (cycloreversion) quantum yields, such as diarylethenes **1**–**4**, are favorable for the localization super-resolution fluorescence microscopy based on single-molecule detection, such as PALM and STORM [35,36,37]. On the other hand, the coordinate-targeted super-resolution fluorescence microscopy, such as RESOLFT microscopy, which claims easy photoswitching of probe molecules from on-states to off-states upon irradiation with a doughnut-shaped low-power laser beam, requires photoswitchable fluorescent molecules with high switching-off (cycloreversion) quantum yields in the order of 10^−3^, such as diarylethene **5** [38,41]. Introducing appropriate alkyl substituents at the reactive carbons would provide a rational molecular design guideline to prepare synthetic molecular probes suitable for super-resolution fluorescence microscopies. 

In conclusion, diarylethenes **1**–**5** with thiophene 1,1-dioxide groups underwent turn-on mode fluorescence switching upon photocyclization reactions. The fluorescent property of the closed-ring isomer depends on the alkyl substituents at the reactive carbons. **2b**–**5b** having ethyl, *n*-propyl, *i*-propyl, and *i*-butyl substituents show higher fluorescence quantum yields than methyl substituted derivative **1b** and maintain the adequate fluorescent property even in polar solvents. It was found that the cycloreversion quantum yield is dramatically increased by introducing *i*-butyl substituents at the reactive carbons.

## 3. Materials and Methods 

Compound **1a** was synthesized according to the previous literature [39]. The syntheses of compounds **2a**–**5a** are described in the supplementary material (Scheme S1). Commercially available reagents for the syntheses were of reagent grade and used without further purification. Solvents for spectral measurements were of spectroscopic grade. 

400 MHz ^1^H NMR spectra were measured with an NMR spectrometer (Bruker, Avance 400). Tetramethylsilane was used as an internal standard. Mass spectrometry was carried out with a mass spectrometer (Shimadzu, GCMS-QP2010Plus) based on electron-impact ionization. 

UV–visible absorption spectra were measured with an absorption spectrophotometer (Hitachi, U-4100). Fluorescence spectra were measured with a fluorescence spectrophotometer (Hitachi, F-2500). No correction was performed on the fluorescence spectra. Fluorescence quantum yields were measured with an absolute PL quantum yield measurement system (Hamamatsu, C9920-02G). The maximum-absorption wavelength of the closed-ring isomer was used as the excitation wavelength for the fluorescence quantum yield measurement.

Photoirradiation for photoreactions was carried out using a 300 W xenon lamp (Asahi spectra, MAX-302). Wavelength of the light was selected using band-pass or cut-off optical filters and a monochromator (Ritsu, MC-10N). Cyclization quantum yields of **1a**–**5a** were determined using 1,2-bis(2,5-dimethyl-3-thienyl)perfluorocyclopentene [42] as a reference. Cycloreversion quantum yields of **1b**–**5b** were determined using 1,2-bis(2-ethyl-6-phenyl-1-benzothiophene-1,1-dioxide-3-yl)perfluorocyclopentene [33,34] as a reference.

## Figures and Tables

**Table 1:** Photophysical and photochemical properties of **1**–**5** in 1,4-dioxane.

	Open-Ring Isomer, a		Closed-Ring Isomer, b		
	*λ*_max_/nm (ε/10^4^ M^−1^ cm^−1^)	Φ_oc_	*λ*_max_/nm (ε/10^4^ M^−1^ cm^−1^)	Φ_co_	Φ_f_
**1**	296 (0.79)	0.14	424 (2.4)	3.5 × 10^−5^	0.07
**2**	297 (0.79)	0.09	433 (2.2)	3.8 × 10^−5^	0.42
**3**	298 (0.80)	0.10	434 (2.1)	7.1 × 10^−5^	0.42
**4**	301 (0.79)	0.21	427 (1.8)	2.6 × 10^−5^	0.42
**5**	299 (0.83)	0.12	438 (2.1)	2.1 × 10^−3^	0.50

*λ*_max_: absorption maximum, *ε*: molar absorption coefficient, Φ_oc_: cyclization quantum yield under irradiation with 313 nm light, Φ_co_: cycloreversion quantum yield under irradiation with 450 nm light, Φ_f_: fluorescence quantum yield under irradiation at the absorption maximum.

## Supplementary Materials

The following are available online at www.mdpi.com/1996-1944/10/9/1021/s1, Table S1: Fluorescence quantum yields of **1b**–**5b** in various solvents, Scheme S1: Syntheses of compounds **2a**–**5a**.

## References

[1] Heilemann M., Dedecker P., Hofkens J., Sauer M. (2009). Photoswitches: Key molecules for subdiffraction-resolution fluorescence imaging and molecular quantification. Laser Photon. Rev..

[2] Raymo F.M. (2013). Photoactivatable synthetic fluorophores. Phys. Chem. Chem. Phys..

[3] Fürstenberg A., Heilemann M. (2013). Single-molecule localization microscopy-near molecular spatial resolution in light microscopy with photoswitchable fluorophores. Phys. Chem. Chem. Phys..

[4] Fukaminato T. (2011). Single-molecule fluorescence photoswitching: Design and synthesis of photoswitchable fluorescent molecules. J. Photochem. Photobiol. C.

[5] Zhang J., Qi Z., Tian H. (2013). Photochromic materials: More than meets the eye. Adv. Mater..

[6] Irie M., Fukaminato T., Sasaki T., Tamai N., Kawai T. (2002). Organic chemistry: A digital fluorescent molecular photoswitch. Nature.

[7] Fukaminato T., Sasaki T., Kawai T., Tamai N., Irie M. (2004). Digital photoswitching of fluorescence based on the photochromism of diarylethene derivatives at a single-molecule level. J. Am. Chem. Soc..

[8] Fukaminato T., Umemoto T., Iwata Y., Yokojima S., Yoneyama M., Nakamura S., Irie M. (2007). Photochromism of diarylethene single molecules in polymer matrices. J. Am. Chem. Soc..

[9] Fukaminato T., Tanaka M., Doi T., Tamaoki N., Katayama T., Mallick A., Ishibashi Y., Miyasaka H., Irie M. (2010). Fluorescence photoswitching of a diarylethene-perylenebisimide dyad based on intramolecular electron transfer. Photochem. Photobiol. Sci..

[10] Fukaminato T., Doi T., Tamaoki N., Okuno K., Ishibashi Y., Miyasaka H., Irie M. (2011). Single-molecule fluorescence photoswitching of a diarylethene-perylenebisimide dyad: Non-destructive fluorescence readout. J. Am. Chem. Soc..

[11] Golovkova T.A., Kozlov D.V., Neckers D.C. (2005). Synthesis and properties of novel fluorescent switches. J. Org. Chem..

[12] Bossi M., Belov V., Polyakova S., Hell S.W. (2006). Reversible red fluorescent molecular switches. Angew. Chem. Int. Ed..

[13] Yan S.F., Belov V.N., Bossi M.L., Hell S.W. (2008). Switchable fluorescent and solvatochromic molecular probes based on 4-amino-n-methylphthalimide and a photochromic diarylethene. Eur. J. Org. Chem..

[14] Berberich M., Krause A.-M., Orlandi M., Scandola F., Würthner F. (2008). Toward fluorescent memories with nondestructive readout: Photoswitching of fluorescence by intramolecular electron transfer in a diaryl ethene-perylene bisimide photochromic system. Angew. Chem. Int. Ed..

[15] Berberich M., Natali M., Spenst P., Chiorboli C., Scandola F., Würthner F. (2012). Nondestructive photoluminescence read-out by intramolecular electron transfer in a perylene bisimide-diarylethene dyad. Chem.-Eur. J..

[16] Berberich M., Würthner F. (2012). Terrylene bisimide-diarylethene photochromic switch. Chem. Sci..

[17] Hell S.W. (2007). Far-field optical nanoscopy. Science.

[18] Hell S.W. (2009). Microscopy and its focal switch. Nat. Meth..

[19] Betzig E., Patterson G.H., Sougrat R., Lindwasser O.W., Olenych S., Bonifacino J.S., Davidson M.W., Lippincott-Schwartz J., Hess H.F. (2006). Imaging intracellular fluorescent proteins at nanometer resolution. Science.

[20] Rust M.J., Bates M., Zhuang X. (2006). Sub-diffraction-limit imaging by stochastic optical reconstruction microscopy (STORM). Nat. Meth..

[21] Bates M., Huang B., Dempsey G.T., Zhuang X.W. (2007). Multicolor super-resolution imaging with photo-switchable fluorescent probes. Science.

[22] Jeong Y.-C., Yang S.I., Ahn K.-H., Kim E. (2005). Highly fluorescent photochromic diarylethene in the closed-ring form. Chem. Commun..

[23] Jeong Y.-C., Yang S.I., Kim E., Ahn K.-H. (2006). Development of highly fluorescent photochromic material with high fatigue resistance. Tetrahedron.

[24] Jeong Y.-C., Yang S.I., Kim E., Ahn K.-H. (2006). A high-content diarylethene photochromic polymer for an efficient fluorescence modulation. Macromol. Rapid Commun..

[25] Jeong Y.-C., Park D.G., Lee I.S., Yang S.I., Ahn K.-H. (2009). Highly fluorescent photochromic diarylethene with an excellent fatigue property. J. Mater. Chem..

[26] Uno K., Niikura H., Morimoto M., Ishibashi Y., Miyasaka H., Irie M. (2011). In situ preparation of highly fluorescent dyes upon photoirradiation. J. Am. Chem. Soc..

[27] Takagi Y., Kunishi T., Katayama T., Ishibashi Y., Miyasaka H., Morimoto M., Irie M. (2012). Photoswitchable fluorescent diarylethene derivatives with short alkyl chain substituents. Photochem. Photobiol. Sci..

[28] Gillanders F., Giordano L., Diaz S.A., Jovin T.M., Jares-Erijman E.A. (2014). Photoswitchable fluorescent diheteroarylethenes: Substituent effects on photochromic and solvatochromic properties. Photochem. Photobiol. Sci..

[29] Sumi T., Kaburagi T., Morimoto M., Une K., Sotome H., Ito S., Miyasaka H., Irie M. (2015). Fluorescent photochromic diarylethene that turns on with visible light. Org. Lett..

[30] Wu T., Johnsen B., Qin Z., Morimoto M., Baillie D., Irie M., Branda N.R. (2015). Two-colour fluorescent imaging in organisms using self-assembled nano-systems of upconverting nanoparticles and molecular switches. Nanoscale.

[31] Bälter M., Li S., Morimoto M., Tang S., Hernando J., Guirado G., Irie M., Raymo F.M., Andréasson J. (2016). Emission color tuning and white-light generation based on photochromic control of energy transfer reactions in polymer micelles. Chem. Sci..

[32] Morimoto M., Kashihara R., Mutoh K., Kobayashi Y., Abe J., Sotome H., Ito S., Miyasaka H., Irie M. (2016). Turn-on mode fluorescence photoswitching of diarylethene single crystals. CrystEngComm.

[33] Takagi Y., Morimoto M., Kashihara R., Fujinami S., Ito S., Miyasaka H., Irie M. (2017). Turn-on mode fluorescent diarylethenes: Control of the cycloreversion quantum yield. Tetrahedron.

[34] Morimoto M., Irie M., Yokoyama Y., Nakatani K. (2017). Turn-on mode fluorescent diarylethenes. Photon-Working Switches.

[35] Nevskyi O., Sysoiev D., Oppermann A., Huhn T., Wöll D. (2016). Nanoscopic visualization of soft matter using fluorescent diarylethene photoswitches. Angew. Chem. Int. Ed..

[36] Arai Y., Ito S., Fujita H., Yoneda Y., Kaji T., Takei S., Kashihara R., Morimoto M., Irie M., Miyasaka H. (2017). One-colour control of activation, excitation and deactivation of a fluorescent diarylethene derivative in super-resolution microscopy. Chem. Commun..

[37] Roubinet B., Weber M., Shojaei H., Bates M., Bossi M.L., Belov V.N., Irie M., Hell S.W. (2017). Fluorescent photoswitchable diarylethenes for biolabeling and single-molecule localization microscopies with optical superresolution. J. Am. Chem. Soc..

[38] Roubinet B., Bossi M.L., Alt P., Leutenegger M., Shojaei H., Schnorrenberg S., Nizamov S., Irie M., Belov V.N., Hell S.W. (2016). Carboxylated photoswitchable diarylethenes for biolabeling and super-resolution RESOLFT microscopy. Angew. Chem. Int. Ed..

[39] Taguchi M., Nakagawa T., Nakashima T., Kawai T. (2011). Photochromic and fluorescence switching properties of oxidized triangle terarylenes in solution and in amorphous solid states. J. Mater. Chem..

[40] Kitagawa D., Sasaki K., Kobatake S. (2011). Correlation between steric substituent constants and thermal cycloreversion reactivity of diarylethene closed-ring isomers. Bull. Chem. Soc. Jpn..

[41] Dedecker P., Hotta J.-I., Flors C., Sliwa M., Uji-i H., Roeffaers M.B.J., Ando R., Mizuno H., Miyawaki A., Hofkens J. (2007). Subdiffraction imaging through the selective donut-mode depletion of thermally stable photoswitchable fluorophores: Numerical analysis and application to the fluorescent protein dronpa. J. Am. Chem. Soc..

[42] Shibata K., Muto K., Kobatake S., Irie M. (2002). Photocyclization/cycloreversion quantum yields of diarylethenes in single crystals. J. Phys. Chem. A.

