# Supplementary material for: Photoswitchable Fluorescent Diarylethene Derivatives with Thiophene 1,1-Dioxide Groups: Effect of Alkyl Substituents at the Reactive Carbons
Source: Materials (Basel). 2017 Sep 2;10(9):1021. doi: 10.3390/ma10091021 (PMC5615676; doi:10.3390/ma10091021)
Supplement: Supplementary file 1 [file materials-10-01021-s001.pdf]

Supplementary Material

Photoswitchable Fluorescent Diarylethene Derivatives with Thiophene 1,1-Dioxide Groups: Effect of Alkyl Substituents at the Reactive Carbons

Masakazu Morimoto [1,*], Takaki Sumi [1] and Masahiro Irie [1,*]

[1] Department of Chemistry and Research Center for Smart Molecules, Rikkyo University, 3-34-1 Nishi-Ikebukuro, Toshima-ku, Tokyo 171-8501, Japan
* Correspondence: m-morimoto@rikkyo.ac.jp, iriem@rikkyo.ac.jp

Table S1 Fluorescence quantum yields of **1b**–**5b** in various solvents.

	**1b**	**2b**	**3b**	**4b**	**5b**
n-Hexane (ε_r = 1.89)	0.31	0.54	0.53	0.38	0.55
1,4-Dioxane (ε_r = 2.22)	0.07	0.42	0.42	0.42	0.50
2MeTHF (ε_r = 6.97)	0.04	0.30	0.30	0.35	0.35
2-Propanol (ε_r = 20.1)	0.03	0.25	0.23	0.34	0.28
Ethanol (ε_r = 25.3)	0.02	0.18	0.19	0.32	0.27

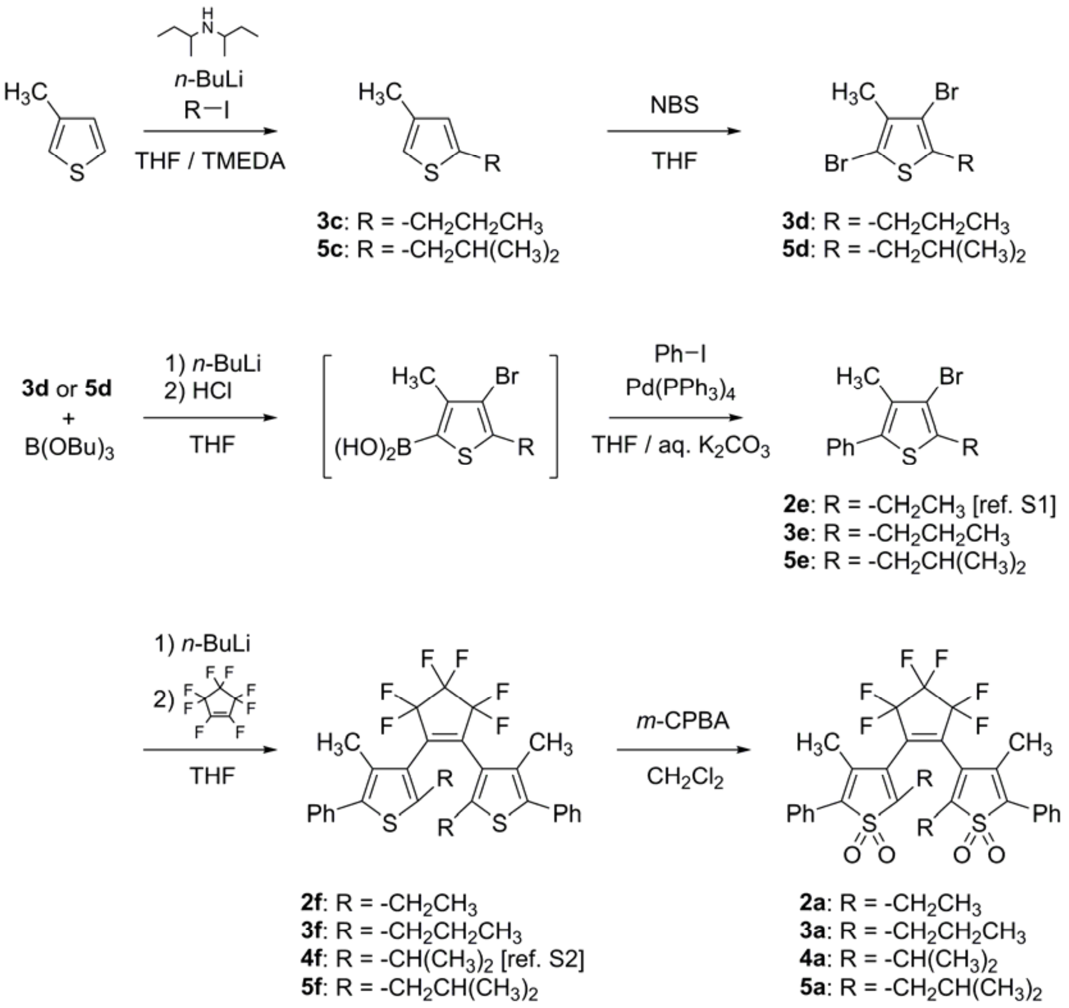

Scheme S1. Syntheses of compounds **2a**–**5a**.

2f

To a dry THF solution (110 mL) containing **2e** [S1] (3.0 g, 11 mmol) was slowly added 1.6 M *n*-BuLi hexane solution (7.6 mL, 12 mmol) at −78°C under a nitrogen atmosphere and the mixture was stirred for 15 min at that temperature. A dry THF solution (10 mL) containing octafluorocyclopentene (0.83 mL, 6.2 mmol) was slowly added at –78°C and the mixture was stirred overnight at that temperature. The reaction was stopped by adding water. The resulting mixture was extracted with ethyl acetate and the organic layer was washed with brine, dried over $MgSO_4$, filtrated, and concentrated. The residue was purified by silica gel column chromatography (hexane) to give **2f** (1.1 g, 35%).

^1H NMR (400 MHz, $CDCl_3$, TMS) δ 0.92 (t, 3H, J = 7.6 Hz), 1.02 (t, 3H, J = 7.6 Hz), 2.08 (d, 3H, J = 1.6 Hz), 2.16 (d, 3H, J = 2.4 Hz), 2.41-2.73(m, 4H), 7.27-7.43 (m, 10H); MS (EI) *m*/*z* 576 $[M]^+$.

2a

To a CH_2Cl_2 solution (30 mL) containing **2f** (43 mg, 0.075 mmol) was added 77% *m*-chloroperbenzoic acid (0.51 g, 2.3 mmol) and the mixture was stirred for 4 days at room temperature. The resulting mixture was treated with aqueous $Na_2S_2O_3$ and extracted with $CHCl_3$. The organic layer was washed with brine, dried over $MgSO_4$, filtrated, and concentrated. The residue was purified by preparative thin-layer silica gel column chromatography (hexane : ethyl acetate = 3 : 1) to give **2a** (27 mg, 56%).

^1H NMR (400 MHz, $CDCl_3$, TMS) δ 1.38-1.43 (m, 6H), 1.89 (s, 4H), 1.97 (s, 2H), 2.47-2.74 (m, 4H), 7.44-7.55 (m, 10H); MS (EI) *m*/*z* 640 $[M]^+$.

4-Methy-2-propylthiophene (3c)

To a dry THF solution (180 mL) containing di-*sec*-butylamine (9.8 mL, 57 mmol) and *N*,*N*,*N'*,*N'*-tetramethylethylenediamine (8.5 mL, 57 mmol) was slowly added 1.6 M *n*-BuLi hexane solution (36 mL, 58 mmol) at −78°C under a nitrogen atmosphere and the mixture was stirred for 30 min at that temperature. After warmed up to 0°C, the mixture was stirred for 30 min. After cooled down to −78°C again, 3-methylthiophene (5.0 mL, 52 mmol) was slowly added and the mixture was stirred for 1 hour. 1-Iodopropane (5.6 mL, 57 mmol) was slowly added and the mixture was stirred for 30 min. After warmed up to room temperature, the mixture was stirred for 16 hours and then dilute HCl was added. The resulting mixture was extracted with diethyl ether and the organic layer was washed with brine, dried over $MgSO_4$, filtrated, and concentrated. The residue was purified by silica gel column chromatography (hexane) to give **3c** (4.3 g, 59%).

^{1}H NMR (400 MHz, $CDCl_3$, TMS) δ 0.97 (t, 3H, *J* = 7.6 Hz), 1.67 (sext, 2H, *J* = 7.6 Hz), 2.73 (t, 2H, *J* = 7.6 Hz), 6.58 (s, 1H), 6.66 (m, 1H); MS (EI) *m*/*z* 140 $[M]^+$.

2,4-Dibromo-3-methyl-5-propylthiophene (3d)

To a THF solution (200 mL) containing **3c** (4.2 g, 30 mmol) was added *N*-bromosuccinimide (12 g, 67 mmol) at 0°C and the mixture was stirred overnight. The resulting mixture was treated with aqueous $Na_2S_2O_3$ and extracted with ethyl acetate. The organic layer was washed with brine, dried over $MgSO_4$, filtrated, and concentrated. The residue was purified by silica gel column chromatography (hexane) to give **3d** (6.3 g, 70%).

^{1}H NMR (400 MHz, $CDCl_3$, TMS) δ 0.98 (t, 3H, *J* = 7.6 Hz), 1.65 (sext, 2H, *J* = 7.6 Hz), 2.17 (s, 3H), 2.71 (t, 2H, *J* = 7.6 Hz); MS (EI) *m*/*z* 296 $[M]^+$, 298 $[M+2]^+$, 300 $[M+4]^+$.

3-Bromo-4-methy-5-phenyl-2-propylthiophene (3e)

To a dry THF solution (250 mL) containing **3d** (9.0 g, 30 mmol) and tributyl borate (8.9 mL, 33 mmol) was slowly added 1.6 M *n*-BuLi hexane solution (21 mL, 34 mmol) at –78°C under a nitrogen atmosphere and the mixture was stirred for 1 hour at that temperature. After warmed up to –20°C, dilute HCl was added. After warmed up to room temperature, $Pd(PPh_3)_4$ (1.7 g, 1.5 mmol), iodobenzene (3.7 mL, 33 mmol), and saturated aqueous K_2CO_3 (30 mL) was added and the mixture was refluxed for 7 hours. The resulting mixture was extracted with ethyl acetate and the organic layer was washed with brine, dried over $MgSO_4$, filtrated, and concentrated. The residue was purified by silica gel column chromatography (hexane) to give **3e** (6.4 g, 73%).

^{1}H NMR (400 MHz, $CDCl_3$, TMS) δ 1.02 (t, 3H, *J* = 7.6 Hz), 1.71 (sext, 2H, *J* = 7.6 Hz), 2.27 (s, 3H), 2.79 (t, 2H, *J* = 7.6 Hz), 7.31-7.41 (m, 5H); MS (EI) *m*/*z* 294 $[M]^+$, 296 $[M+2]^+$.

3f

To a dry THF solution (70 mL) containing **3e** (2.0 g, 6.8 mmol) was slowly added 1.6 M *n*-BuLi hexane solution (4.7 mL, 7.5 mmol) at –78°C under a nitrogen atmosphere and the mixture was stirred for 10 min at that temperature. A dry THF solution (10 mL) containing octafluorocyclopentene (0.54 mL, 4.0 mmol) was slowly added at –78°C and the mixture was stirred overnight at that temperature. The reaction was stopped by adding water. The resulting mixture was extracted with ethyl acetate and the organic layer was washed with brine, dried over $MgSO_4$, filtrated, and concentrated. The residue was purified by silica gel column chromatography (hexane) to give **3f** (1.0 g, 49%).

^1H NMR (400 MHz, $CDCl_3$, TMS) δ 0.92 (t, 3H, *J* = 7.6 Hz), 1.02 (t, 3H, *J* = 7.6 Hz), 1.46-1.77 (m, 4H), 2.08 (d, 3H, *J* = 1.6 Hz), 2.16 (d, 3H, *J* = 2.8 Hz), 2.41-2.72 (m, 4H), 7.27-7.42 (m, 10H); MS (EI) *m*/*z* 604 $[M]^+$.

3a

To a CH_2Cl_2 solution (250 mL) containing **3f** (1.4 g, 2.3 mmol) was added 77% *m*-chloroperbenzoic acid (12 g, 53 mmol) and the mixture was stirred for 3 days at room temperature. The resulting mixture was treated with aqueous $Na_2S_2O_3$ and extracted with $CHCl_3$. The organic layer was washed with aqueous NaOH and brine, dried over $MgSO_4$, filtrated, and concentrated. The residue was purified by silica gel column chromatography ($CHCl_3$) to give **3a** (1.0 g, 65%).

^1H NMR (400 MHz, $CDCl_3$, TMS) δ 1.01-1.06 (m, 6H), 1.72-2.04 (m, 4H), 1.89 (s, 3.8H), 1.95 (s, 2.2H), 2.27-2.66 (m, 4H), 7.43-7.55 (m, 10H); MS (EI) *m*/*z* 668 $[M]^+$.

4a

To a CH_2Cl_2 solution containing **4f** [S2] (0.53 g, 0.88 mmol) was added 77% *m*-chloroperbenzoic acid (4.0 g, 18 mmol) and the mixture was stirred for 3 days at room temperature. The resulting mixture was treated with aqueous K_2CO_3 and aqueous $Na_2S_2O_3$ and extracted with $CHCl_3$. The organic layer was washed with brine, dried over $MgSO_4$, filtrated, and concentrated. The residue was purified by silica gel column chromatography ($CHCl_3$) to give **2a** (32 mg, 5%).

^1H NMR (400 MHz, $CDCl_3$, TMS) δ 1.39 (d, 5H, *J* = 6.8 Hz), 1.47 (d, 5H, *J* = 6.8 Hz), 1.51 (d, 2H, *J* = 6.8 Hz), 1.93 (d, 1.5H, *J* = 2.4 Hz), 2.02 (d, 4.5H, *J* = 3.2 Hz), 2.74-2.81 (m, 2H), 7.46-7.55 (m, 10H); MS (EI) *m*/*z* 668 $[M]^+$.

2-Isobutyl-4-methylthiophene (5c)

To a dry THF solution (180 mL) containing di-*sec*-butylamine (9.8 mL, 57 mmol) and *N*,*N*,*N'*,*N'*-tetramethylethylenediamine (8.5 mL, 57 mmol) was slowly added 1.6 M *n*-BuLi hexane solution (36 mL, 58 mmol) at –78°C under a nitrogen atmosphere and the mixture was stirred for 1 hour at that temperature. 3-Methylthiophene (5.0 mL, 52 mmol) was slowly added and the mixture was stirred for 1 hour. 1-Iodo-2-methylpropane (7.4 mL, 64 mmol) was slowly added and the mixture was stirred for 1 hour. After warmed up to room temperature, the mixture was stirred overnight and then dilute HCl was added. The resulting mixture was extracted with diethyl ether and the organic layer was washed with brine, dried over $MgSO_4$, filtrated, and concentrated. The residue was purified by silica gel column chromatography (hexane) to give **5c** (2.3 g, 29%).

^{1}H NMR (400 MHz, $CDCl_3$, TMS) δ 0.94 (d, 6H, *J* = 7.6 Hz), 1.86 (m, 1H), 2.21 (s, 3H), 2.62 (dd, 2H, *J* = 7.2 Hz, 0.8 Hz), 6.56 (s, 1H), 6.67 (m, 1H); MS (EI) *m*/*z* 154 $[M]^+$.

2,4-Dibromo-5-isobutyl-3-methylthiophene (5d)

To a THF solution (140 mL) containing **5c** (2.1 g, 14 mmol) was added *N*-bromosuccinimide (5.3 g, 30 mmol) at 0°C and the mixture was stirred overnight. The resulting mixture was treated with aqueous $Na_2S_2O_3$ and extracted with ethyl acetate. The organic layer was washed with brine, dried over $MgSO_4$, filtrated, and concentrated. The residue was purified by silica gel column chromatography (hexane) to give **5d** (1.6 g, 36%).

^{1}H NMR (400 MHz, $CDCl_3$, TMS) δ 0.95 (d, 6H, *J* = 6.8 Hz), 1.92 (m, 1H), 2.18 (s, 3H), 2.62 (d, 2H, *J* = 7.2 Hz); MS (EI) *m*/*z* 310 $[M]^+$, 312 $[M+2]^+$, 314 $[M+4]^+$.

3-Bromo-2-isobutyl-4-methyl-5-phenylthiophene (5e)

To a dry THF solution (45 mL) containing **5d** (1.4 g, 4.5 mmol) and tributyl borate (1.2 mL, 4.5 mmol) was slowly added 1.6 M *n*-BuLi hexane solution (3.1 mL, 5.0 mmol) at –78°C under a nitrogen atmosphere and the mixture was stirred for 1 hour at that temperature. After warmed up to room temperature, dilute HCl was added. $Pd(PPh_3)_4$ (0.26 g, 0.22 mmol), iodobenzene (0.50 mL, 4.5 mmol), and saturated aqueous K_2CO_3 (20 mL) was added and the mixture was refluxed for 7 hours. The resulting mixture was extracted with ethyl acetate and the organic layer was washed with brine, dried over $MgSO_4$, filtrated, and concentrated. The residue was purified by silica gel column chromatography (hexane) to give **5e** (0.73 g, 53%).

^{1}H NMR (400 MHz, $CDCl_3$, TMS) δ 1.00 (d, 6H, *J* = 6.8Hz), 2.00 (m, 1H), 2.27 (s, 3H), 2.71 (d, 2H, *J* = 7.2 Hz), 7.29-7.42 (m, 5H); MS (EI) *m*/*z* 308 $[M]^+$, 310 $[M+2]^+$.

5f

To a dry THF solution (20 mL) containing **5e** (0.60 g, 1.9 mmol) was slowly added 1.6 M *n*-BuLi hexane solution (1.3 mL, 2.1 mmol) at –78°C under a nitrogen atmosphere and the mixture was stirred for 15 min at that temperature. A dry THF solution (5 mL) containing octafluorocyclopentene (0.14 mL, 1.0 mmol) was slowly added at –78°C and the mixture was stirred for 1 hour at that temperature. After warmed up to room temperature, the mixture was stirred for 5 hours. The reaction was stopped by adding water. The resulting mixture was extracted with diethyl ether and the organic layer was washed with brine, dried over $MgSO_4$, filtrated, and concentrated. The residue was purified by silica gel column chromatography (hexane) to give **5f** (0.20 g, 34%).

^1H NMR (400 MHz, $CDCl_3$, TMS) δ 0.86 (m, 7H), 0.95 (d, 3H, *J* = 6.8 Hz), 1.04 (d, 2H, *J* = 6.8 Hz), 1.82-2.01 (m, 2H), 2.05-2.07 (m, 2.6H), 2.20-2.30 (m, 4.5H), 2.38-2.60 (m, 2.9H), 7.28-7.45 (m, 10H); MS (EI) *m*/*z* 632 $[M]^+$.

5a

To a CH_2Cl_2 solution (25 mL) containing **5f** (0.16 g, 0.25 mmol) was added 77% *m*-chloroperbenzoic acid (1.7 g, 7.6 mmol) and the mixture was stirred for 2 days at room temperature. The resulting mixture was treated with aqueous K_2CO_3 and aqueous $Na_2S_2O_3$ and extracted with $CHCl_3$. The organic layer was washed with brine, dried over $MgSO_4$, filtrated, and concentrated. The residue was purified by silica gel column chromatography ($CHCl_3$) and HPLC (Wakosil 5SIL ϕ20 mm × 250 mm, hexane : ethyl acetate = 9 : 1, 10 mL/min, 313 nm) to give **5a** (0.10 g, 56%).

^1H NMR (400 MHz, $CDCl_3$, TMS) δ 0.97-1.08 (m, 12H), 1.92 (d, 3H, *J* = 2.0 Hz), 1.97 (d, 3H, *J* = 1.6 Hz), 2.03-2.21 (m, 2H), 2.44-2.75 (m, 4H), 7.45-7.55 (m, 10H); MS (EI) *m*/*z* 696 $[M]^+$.

References

S1 Kanazawa, R.; Taguchi, M.; Nakashima, T.; Kawai, T. *New J. Chem.* **2015**, *39*, 7397-7402.

S2 Uchida, K.; Guillaumont, D.; Tsuchida, E.; Mochizuki, G.; Irie, M.; Murakami, A.; Nakamura, S. *J. Mol. Struct.: THEOCHEM* **2002**, *579*, 115-120.
